# Development of a questionnaire to measure health-related quality of life (HRQoL) in patients with atrial fibrillation (AF-QoL)

**DOI:** 10.1186/1477-7525-5-37

**Published:** 2007-07-04

**Authors:** Xavier Badia, Fernando Arribas, Jose Miguel Ormaetxe, Rafael Peinado, Miguel Sainz de los Terreros

**Affiliations:** 1 IMS Health, Dr. Ferran, 25, 2°, Barcelona 08034, Spain; 2 Hospital 12 de Octubre, Madrid, Spain; 3 Hospital de Basurto, Bilbao, Spain; 4 Hospital La Paz, Madrid, Spain; 5 3M Farmacéutica, Madrid, Spain

## Abstract

**Background:**

The Health-Related Quality of Life (HRQoL) assessment in atrial fibrillation (AF) patients has traditionally been carried out in a poorly standardised fashion, or via the use of non disease-specific HRQoL questionnaires. The development of a HRQoL questionnaire with a good measuring performance will allow for a standardised assessment of the impact of this disease on the patient's daily living.

**Methods:**

A bibliography review was conducted to identify the most relevant domains of daily living in AF patients. Subsequently, a focus group was created with the aid of cardiologists, and 17 patients were interviewed to identify the most-affected HRQoL domains. A qualitative analysis of the interview answers was performed, which was used to develop a pilot questionnaire administered to a 112-patient sample. Based on patient responses, an analysis was carried out following the statistical procedures defined by the Classical Test Theory (CTT) and the Item Response Theory (IRT). Reliablility was assessed via Cronbach's coefficient alpha and item-total score correlations. A factorial analysis was performed to determine the number of domains. For each domain, a Rasch analysis was carried out, in order to reduce and stand hierarchically the questionnaire items.

**Results:**

By way of the bibliography review and the expert focus group, 10 domains were identified. The patient interviews allowed for the identification of 286 items that later were downsized to 40 items. The resultant preliminary questionnaire was administered to a 112-patient sample (pilot study). The Rasch analysis led to the definition of two domains, comprising 7 and 11 items respectively, which corresponded to the psychological and physical domains (18 items total), thereby giving rise to the initial AF-QoL-18 questionnaire. Cronbach's coefficient alpha was acceptable (0.91).

**Conclusion:**

An initial HRQoL questionnaire, AFQoL-18, has been developed to assess HRQoL in AF patients.

## Background

The main objective of the management of atrial fibrillation (AF) patients has been to restore and maintain sinus rhythm, given that palpitations in these patients increase the risk of stroke and the likelihood of left ventricular dysfunction [1]. The goal of antiarrhythmic therapies is to relieve symptoms, prevent complications and reduce disease-related mortality in this population [2]. Other pharmacological therapy options are aimed at maintaining the heart rate under control. Non-pharmacological therapy options include catheter ablation, pacemakers, internal defibrillation and antiarrhythmic surgery, although the impact of such therapies on morbidity and mortality is unknown.

Traditionally, the reduction of morbidity and mortality rates in these patients has been assessed as the main goal of the effectiveness of AF therapy [3,4]. Nevertheless, few studies take into account the impact of the disease and its treatments on patients' health-related quality of life (HRQoL), despite HRQoL assessment's being important for several reasons. In the first place, drug efficacy is becoming increasingly similar among different agents, and often a given therapy is selected based on the potential improvement in the HRQoL, especially in elderly patients. Secondly, patients' active involvement with physicians in therapeutic decision making often focuses on recognizing and choosing the therapeutic option that will have a lesser impact on the patient's daily living. Lastly, regulatory authorities increasingly request data on HRQoL in reports submitted for the approval of new drugs or therapeutic modalities [3].

Some studies have found that the impact is greater on the physical domain, and that HRQoL also depends on individual experiences, beliefs and expectations regarding the disease. Sociodemographic variables, like age and gender, also affect HRQoL, with female patients and those under the age of 69 obtaining the worst score. [5-7].

HRQoL assessment as part of the clinical management of AF will allow the practitioner to identify those aspects often overlooked in clinical practice regarding the patient's perception of the disease and its course, which in turn could help improve control over the disease. It has been observed that restoration of sinus rhythm is the best predictor of the disease's impact on the patient's HRQoL [7]. Studies have shown that the correlation between clinical measurements, like time to first AF recurrence, concurrent stroke or symptoms, and subjective measurements like HRQoL, is poor. The use of specific measurements for HRQoL will allow a better assessment of the impact of this disease on patient's daily living while, in addition, other specific aspects of the disease can be identified. These would significantly improve the patient's HRQoL through strategies aimed at minimizing the impact of AF on HRQoL.

The goal of the present study is to develop a HRQoL-specific questionnaire for AF patients that can be applied to any of the types of AF (paroxysmal or permanent). The project is divided into two phases. The first one focuses on the development of the preliminary specific questionnaire. The second will focus on the development of the final questionnaire, following a prospective trial, and on the assessment of the questionnaire's measurement properties in a large sample of patients.

In this article, the methodology of the first phase of the questionnaire's development is explained.

## Methods

The HRQoL questionnaire in AF patients (AF-QoL-18) was developed in three phases: item generation, item selection and reduction of the items.

### Item generation

Firstly, a bibliography review was conducted to identify the most relevant aspects describing the impact of AF on the patients' HRQoL. That information was used to elaborate a survey for identifying AF-related symptoms as assessed by three cardiologists specialised in arrhythmias. With a list of HRQoL domains related to AF, the experts included each symptom in one of these domains, based on their own clinical practice. Each symptom was assessed in terms of its importance for the patient according to the experts, and the frequency with which it is observed in the physician's office. Finally, these experts took part in a Focus Group [8] where previously identified aspects were discussed and evaluated as necessary in cases of doubt or discrepancies.

This information was used to create a semi-structured interview script to be administered to AF patients, in order to identify which items and domains had the greatest impact on daily living from the patients' point of view. A psychologist specialised in the development of patient-focused scales interviewed a total of 17 AF patients in three Spanish hospitals in Bilbao and Madrid. All respondents agreed to have their interviews recorded on audio. The interviews lasted between 35 and 50 minutes. After transcribing the interviews, relevant phrases/expressions were obtained for each of the assessed domains [9].

### Item selection

An initial qualitative reduction of the identified expressions was carried out, in which expressions considered inappropriate, ambiguous or redundant were excluded. The expressions included were slightly reworded as statements capable of being used as items in an initial questionnaire.

Each expression was assessed at a second experts' meeting, in terms of *clarity *(how easy they were to understand from the patients' perspective), *importance *(specific relevance of the expression for AF patients) and *frequency *(number of cases in which AF patients utilised the expression), by means of a Likert-type scale ranging from 1 (not very clear/frequent/important) to 5 (very clear/frequent/important) [10]. The information was analysed by assessing the degree of concordance among experts for each response scale (clarity, importance and frequency) by means of Cronbach's alpha. A description of the scores obtained on each scale was made by means of basic descriptive statistics (mean, median, standard deviation, minimum value and percentiles for 10 cut-off values, plus 25th and 75th quartiles). Finally, a qualitative reduction of the items ensued, based on the mean scores derived from the experts' responses. Other qualitative aspects were taken into consideration in the item reduction based on the judgment of HRQoL experts. High clarity was defined as a mean score greater than 4.33 (percentile 50); high importance, as a score greater than 4.00 (percentile 75); high frequency, as a mean score greater than 3.00 (50^th ^percentile); high frequency X importance, as a mean score greater than 16.00.

The identified items were edited to create a self-administered preliminary questionnaire for a sample of 112 patients with an AF diagnosis in three Spanish hospitals. Patients received instructions in order to fill in the items properly, as well as the response scales ('totally agree', 'sufficiently agree', 'neither agree nor disagree', 'sufficiently disagree', 'totally disagree'). Data regarding age, sex, date of AF diagnosis and AF type (permanent or paroxysmal) were gathered for each participant in the pilot study.

### Reduction of the items

Based on the patients' responses to the preliminary questionnaire items, an item analysis was performed following a strategy based on the Classic Test Theory (CTT) [1] and the Item Response Theory (IRT) [12], and specifically on the Rasch analysis [13].

By means of CTT statistics analysis, criteria such as item internal consistency (analysis of item-total score correlations) and reliability (Cronbach's alpha) were considered. The combination of items comprising the questionnaire domains was established by means of exploratory factor analysis techniques. A Rasch analysis was performed for each factor selected in order to reduce the questionnaire. The Rasch model specifies that an individual's response to each item represents the result of an interaction between the item's position (calibration) and the person (scores). This model constructs a line of measurement, with the items placed hierarchically, and provides a statistical adjustment indicating to what degree an item describes the group of subjects responding to the questionnaire [14,15]. Rasch analyses were carried out using BIGSTEPS software, version 2.7.3 [14]. The term 'Measure' is a measure of weight or difficulty for each item (items with a negative measure indicate more difficult). Georg Rasch suggests chi-square fit statistics to control the applicability of data to his model (Rasch 1980). The chi-squares in common use are known as OUTFIT and INFIT. These are reported as mean-squares, chi-square statistics divided by their degrees of freedom, so that they have a ratio-scale form with expectation 1 and range 0 to +infinity. INFIT and OUTFIT MNSQ indices > 1.3 were excluded [16]. For each domain analysed, those items with a separation below 1 were eliminated. Successive Rasch analyses were performed until all items in all domains showed an appropriate capability to fit.

After the Rasch analysis, the items that finally made up the reduced version of the questionnaire were numbered.

## Results

### Item generation

Literature on AF and quality of life was reviewed, as well as HRQoL questionnaires administered to AF patients. A search in the MEDLINE database for the previous eight years was conducted to identify topics related to HRQoL and AF. With the consensus of AF experts, the domains identified as having the greatest impact on HRQoL were the physical, psychological, social, daily activities, symptoms, cognitive, perception of health, sexuality, energy/vitality and sleep domains. Although it is not identified in the literature, the "sleep" domain was included, distinguishing between difficulty in falling asleep and sudden awakening due to the onset of tachycardia or palpitations.

### Item selection

Based on the interviews conducted on 17 AF patients, 286 expressions were identified. The number of expressions was reduced to 94, which constituted the item identification questionnaire administered to the experts' group at their second meeting. The analysis of each item's score performed by the three experts showed good observer/expert consistency as regards expression 'importance' and 'frequency' (Cronbach's α 0.73 and 0.80, respectively). After the elimination of items not fulfilling the criteria specified in the "Item selection" section in the "Material and Methods" chapter, a further analysis reduced the total number of items to 40.

### Reduction of the items

The preliminary 40-item questionnaire (henceforth AF-QoL 40) was self-administered to a pilot sample of 112 patients with an AF diagnosis from 3 Spanish hospitals (Table 1). Mean age (SD) was 60.52 years (13.43) with a predominance of male patients (64.3%). 52.7% of the sample presented paroxysmal AF, and the mean time in years (SD) from diagnosis was 3.9 years (3.83) (Table 2).

**Table 1:** Preliminary items in the unreduced AF-QoL questionnaire (AF-QoL-40).

**Due to my arrhythmia (atrial fibrillation)...**
1. I get tired when I climb slopes or stairs.
2. I get more tired than usual when I carry out physical exercise (jogging, playing tennis, swimming, etc.).
3. I have reduced the amount of physical exercise (jogging, playing tennis, swimming, etc.) I perform.
4. I have stopped performing physical exercise (jogging, playing tennis, swimming, etc.).
5. I get tired when I walk for thirty minutes, and I have to rest.
6. I get tired during a brisk walk.
7. I feel palpitations when I run.
8. I get nervous.
9. I have felt depressed since I was diagnosed with the disease.
10. I feel insecure.
11. I am very aware of my heart and my general health.
12. I feel sadder and more depressed than before I was diagnosed with my disease.
13. I get scared when I feel that my heart beats at high speed.
14. I am afraid that something might happen to me while I am alone.
15. I have negative thoughts about my future.
16. I feel depressed when I find that I get tired.
17. I am afraid of having a sudden and unexpected tachycardia.
18. I feel anxious when I have tachycardia.
19. I am afraid that tachycardia will appear again.
20. I feel insecure when I have to travel.
21. I only go to places with hospitals nearby.
22. My disease has an impact on my family life.
23. I find it difficult to get out of the house to carry out any activity.
24. I feel very tired.
25. I feel as if something were pressing against my heart.
26. I feel exhausted and tired during the arrhythmia (tachycardia).
27. I feel a general tiredness after the arrhythmia (tachycardia).
28. I feel depressed when I think that my disease will last forever.
29. I am afraid of pain or of suffering a heart attack.
30. What affects me the most is the helplessness I feel when I suffer a crisis (tachycardia or arrhythmia episode).
31. I feel affected by the impossibility of carrying out certain activities; "I want to, but my body cannot".
32. I am afraid that my disease complicates things.
33. My disease has impaired my quality of life.
34. Changes have occurred in my sexual activity due to the medications I take.
35. Sexual relationships are less frequent than before I was diagnosed with the disease.
36. I get tired during sexual intercourse, and that is a limitation I feel.
37. I am afraid that my heart is "triggered" during sexual intercourse.
38. I feel a lack of vitality/energy to propose activities.
39. I felt more vitality before I was diagnosed with the disease.
40. I wake up at night due to the tachycardia (arrhythmia).

**Table 2:** Sociodemographic and clinical characteristics of the 112-patient sample with AF from three Spanish hospitals

**VARIABLE**	**N (%)**
**Sex**	
Male	72 (64.3)
Female	40 (35.7)
**Age**: mean (SD) (years)	60.52 (13.43)
**Type of AF**	**N (%)**
Permanent AF	53 (47.3)
Paroxysmal AF	59 (52.7)
**Time from diagnosis**: mean (SD) (years)	3.91 (2.07)

AF-QoL 40 feasibility was completely fulfilled by the 85.7% of the sample. It was noted that the highest percentage of unanswered questions was found for items 4, 34, 35, 36 and 37, four of which refer to sexual activity.

As for the internal consistency of the instrument, the Cronbach's alpha for the 40 items was 0.956.

The global score for the preliminary questionnaire AF-QoL-40 ranged from 40 (worse HRQoL) to 200 (best HRQoL). To make data interpretation easier, scores were standardised from 0 (worst HRQoL) to 100 (best HRQoL) following this formula:

P _100 _= [100/(p _max_-p_min_)]*(p_real _- p_min_)

With the standardised scores, the mean (SD) was 39.4 (20.5) points.

Correlations between each item and the global score ranged from 0.4 (item 2) to 0.76 (item 14). No item was excluded based on correlations below 0.4.

A factorial analysis of the 40 items was conducted, and two factors were clearly identified that, in conjunction, accounted for 50.2% of the variance explained, the first factor had a variance explained of 37.8%, and of 12.3% the second factor, there was a third factor with only 5.7% of variance explained. The first factor consists of 21 items and the second, of 19 items. Item allocation to either of the two factors is done when the item burden is above 0.4 in any of the two factors. No item was excluded for not fulfilling this criterion.

Subsequently, a Rasch analysis was performed for each of the two factors in order to reduce the number of questionnaire items. For the first factor, the number of items was reduced from 21 to 7 items (henceforth AF-QoL-7) and the range of locations for this items was -0.70 to 0.59 and for the second, from 19 to 11 items (henceforth AF-QoL-11) and the range of locations was greater, -0.87 to 0.72 (Table 3). For both factors, reliability was 0.82 and separation was above 2 points.

**Table 3:** Summary of the RASCH analysis for each factor

	**Rasch analysis for factor # 1 Psychological Domain (AF-QoL-7)**				
**AF-QoL-40 Item Numer**	**Item**	**Measure**	**Error**	**Infit**	**Outfit**
30	What affects me the most is the helplessness I feel during a crisis	0.59	0.13	1.09	1.07
32	I am afraid that my disease complicates things.	0.34	0.12	1.09	1.05
29	I am afraid of pain or of suffering a heart attack.	0.13	0.12	0.98	0.92
17	I am afraid of having a sudden and unexpected tachycardia.	-0.02	0.12	0.96	0.99
16	I feel depressed when I find that I get tired.	-0.05	0.12	1.08	1.09
28	I feel depressed when I think that my disease will last forever.	-0.29	0.11	0.79	0.76
15	I have negative thoughts about my future.	-0.70	0.11	1.12	1.03
	**Rasch analysis for factor # 2 Physical Domain (AF-QoL-11)**				

2	I get more tired than usual when I perform physical exercise	0.72	0.12	1.24	1.17
6	I get tired during a brisk walk.	0.62	0.11	0.94	0.76
39	I felt more vitality before I was diagnosed with the disease.	0.48	0.11	0.98	0.97
4	I have stopped performing physical exercise	0.15	0.10	1.17	1.08
33	My disease has impaired my quality of life.	0.14	0.10	0.97	0.89
31	I feel affected by the impossibility of carrying out certain activities; "I want to, but my body cannot".	0.01	0.10	0.81	0.73
35	Sexual relationships are less frequent than before I was diagnosed with the disease.	-0.13	0.10	0.96	0.89
5	I get tired when I walk for thirty minutes and I have to rest.	-0.16	0.09	1.24	1.06
34	Changes have occurred in my sexual activity due to the medications I take.	-0.40	0.09	0.97	1.09
37	I am afraid that my heart is "triggered" during sexual intercourse.	-0.56	0.09	1.07	1.10
23	I find it difficult to get out of the house to carry out any activity.	-0.87	0.09	1.01	1.05

Based on the content of the items included in each factor, it can be said that the AF-QoL-7 items deal with the psychological domain while those of the AF-QoL-11 deal with physical activity. The questionnaire comprising the AF-QoL-7 and the AF-QoL-11 domains is identified as AF-QoL-18.

Internal consistency (Cronbach's α) for AF-QoL-18 and each of the domains was appropriate, with values of 0.91 for the global questionnaire and 0.89 and 0.90 for AF-QoL-7 and AF-QoL-11, respectively.

The mean score (SD) for both AF-QoL-18 and each of its domains was similar (ranging from 39.6 to 38.03), and slightly lower (worse HRQoL) for the physical domain (AF-QoL-11).

Finally, the reduced version of the questionnaire (AF-QoL-18) and the 40-item unreduced version (AF-QoL-40) were compared; correlations were above 0.80 both for the original instrument as well as for each of the reduced domains. On the one hand, each factor showed a high correlation with the AF-QoL-18 questionnaire global score, while correlation between both factors/domains was lower (0.51) (Table 4). Table 5 shows a summary of the most relevant outcomes obtained for the initial 40-item version and the reduced 18-item one with its domains/factors.

**Table 4:** Correlation between AF-Qol-18, AF-QoL-7 (psychological) and AF-QoL-11(physical)

	AF-Qol 18	AF-Qol7 **(psychological)**	AF-Qol11 **(physical)**
AF-Qol7 **(psychological)**	r = 0.813 **p = 0.000** **n = 97**		
AF-Qol11 **(physical)**	r = 0.904 **p = 0.000** **n = 97**	r = 0.514 **p = 0.000** **n = 97**	
AF-Qol40 (initial)	r = 0.944 **p = 0.000** **n = 96**	r = 0.848 **p = 0.000** **n = 96**	r = 0.802 **p = 0.000** **n = 96**

**Table 5:** Summary of the measurement properties

	**AF-Qol40^1^**	**AF-Qol18^2^**	**AF-Qol7^3^**	**AF-Qol11^4^**
N° items	40	18	7	11
**Score distribution**				
Observations	96	97	106	97
Mean	39.41	38.87	39.59	38.03
SD	20.48	21.53	26.58	23.69
Percentile 50	36.56	37.50	33.93	36.36
% score 0	1 (0.9%)	3 (2.7%)	4 (3.6%)	4 (3.6)
% score 100	0	0	2 (1.8%)	0
Item total correlation	0.40–0.76	0.45–0.76	0.74–0.85	0.56–0.76
Reliability	0.956	0.915	0.901	0.891
**Rasch analysis**				
Person separation	3.89	2.55	2.12	2.17
Person reliability	0.94	0.87	0.82	0.82

^1 ^Preliminary and unreduced questionnaire

^2 ^Reduced questionnaire after the Rasch analysis

^3 ^Psychological domain items

^4 ^Physical domain items

The analysis of the AF-QoL-18 questionnaire scores and its domains in terms of age and sex did not show statistically significant correlations, although it was observed that women's scores were slightly lower than men's (worse HRQoL) in the physical domain (AF-QoL-11), and slightly higher (better HRQoL) in the psychological domain and the global score (AF-QoL-7 and AF-QoL-18). Likewise, in terms of time (in years) from AF diagnosis, no significant correlations were observed for the questionnaire or with either of the two domains. However, statistically significant differences were observed (p < 0.01) in the psychological domain (AF-QoL-7) between patients with paroxysmal AF and permanent AF; impact on HRQoL was higher in patients with paroxysmal AF. No statistically significant differences were found for the global questionnaire scores (AF-QoL-18) or the physical domain (AF-QoL-11).

The results obtained in terms of number of items, properties of cross-section measurement and relationship with sociodemographic and clinical variables will be further assessed in the prospective validation study (second phase of the project).

## Discussion

The present article deals with the development of a specific questionnaire designed to assess HRQoL in AF patients. The main advantage of a specific questionnaire with respect to a generic instrument is that the former allows one to assess domains relevant to and exclusive of AF, and sensitivity to changes in health status is greater.

In the questionnaire development phase, methodology based both on CTT and IRT was used, thus yielding complementary results that reinforce the robustness of the data. Other authors have combined both methodologies as well in the development of questionnaires, with equally positive results [17,18].

A limitation in published studies is the poorly standardized methodology for assessing HRQoL in AF patients or the administration of generic HRQoL questionnaires. The idea of developing a specific questionnaire based on systematic methodology arose from the absence of HRQoL questionnaires exclusive for AF in the literature [19].

Little is known about the impact of AF on daily living in less severe or asymptomatic cases, since most studies have assessed HRQoL on symptomatic patients who are intolerant or refractory to antiarrhythmic therapy or in those treated with ablation. This is due to the fact that oftentimes HRQoL was assessed in the context of clinical trials. Other general limitations of the studies assessing HRQoL are the reduced sample size of the study populations; the fact that most designs do not include a control group (given the importance of distinguishing the impact of HRQoL in AF patients from that of patients with other cardiac diseases); and the bias produced, in many cases, by questionnaires that are not self-administered by the patients [3].

Assessment of HRQoL in these patients through a specific questionnaire will allow to study whether differences in patient gender and age exist, as has been observed in previous studies [20]. The relationship between clinical variables and HRQoL will also be assessed more accurately, since the poor relationship between these two variables has been found to be potentially grounded in that patients' perceptions do not depend on objective measurements of AF, by some authors [21]. A clear example is the poor correlation between left ventricular dysfunction and NYHA functional class [22]. The use of a specific questionnaire for AF will be suitable tool for detecting aspects in patients' lives that most affect their HRQoL and for elaborating strategies to minimize their impact.

Current results seem to point out that the psychological impact may be greater in patients with paroxysmal AF than in patients with permanent AF, though the questionnaire's global scores are similar in both groups. Scores obtained indicate a negative impact in HRQoL regardless of the type of AF. The results of the second stage of this project will confirm or provide more insight on the negative impact of AF in patients' HRQoL, as has been observed in different studies referenced in this article and the results obtained in the preliminary validation of the AF-QoL-18 Study. In the first stage of the project, the results have been found to be coherent with those of other studies. In such studies, women seem to have a worse HRQoL, but the AF-Qol-18 has shown signs that the impact on women is greater in the physical domain than in the psychological domain. However, this aspect, as has already been commented on, will be better established in the second stage of this project, when a larger number of patients will be assessed.

## Conclusion

To conclude, the preliminary results of the AF-QoL-18 questionnaire seem to indicate that it can be applied in daily clinical practice and in the clinical research context. An assessment of the remainder of the properties, such as test-retest reliability, longitudinal validity and the sensitivity to the change of the AF-QoL-18 questionnaire, is required to establish and assess the impact that the disease and its treatment have on HRQoL.

It is not to be disregarded that once the validation stage is completed, the AF-QoL-18 feasibility will improve, since the Rasch analysis will be repeated to verify or change any of the 18 items that were initially proposed.

## Authors' contributions

All the authors have read and approved the final manuscript.

XB: took part in all the steps for the design and development of the questionnaire, as well in the writing of the paper.

FA: participated giving scientific advice as an expert on atrial fibrillation, as well as in the process of the questionnaire development. Collaborated in the writing of the paper.

JMO: participated giving scientific advice as an expert on atrial fibrillation, as well as in the process of the questionnaire development. Collaborated in the writing of the paper.

RP: participated giving scientific advice as an expert on atrial fibrillation, as well as in the process of the questionnaire development. Collaborated in the writing of the paper.

MS: took part in all the steps for the design and development of the questionnaire, as well in the writing of the paper.
